# Exploring researchers’ experiences of working with a researcher-driven, population-specific community advisory board in a South African schizophrenia genomics study

**DOI:** 10.1186/s12910-015-0037-5

**Published:** 2015-07-02

**Authors:** Megan M. Campbell, Ezra Susser, Jantina de Vries, Adam Baldinger, Goodman Sibeko, Michael M. Mndini, Sibonile G. Mqulwana, Odwa A. Ntola, Raj S. Ramesar, Dan J. Stein

**Affiliations:** Department of Psychiatry and Mental Health, University of Cape Town, J-Block, Groote Schuur Hospital, Observatory, Cape Town, South Africa; Mailman School of Public Health, Columbia University and New York State Psychiatric Institute, New York, NY USA; Department of Medicine, University of Cape Town, Cape Town, South Africa; MRC Human Genetics Research Unit, Division of Human Genetics, Institute of Infectious Disease and Molecular Medicine, Department of Clinical Laboratory Science, University of Cape Town, Cape Town, South Africa

**Keywords:** Community engagement, Genomics research, Community advisory board, Schizophrenia, Xhosa

## Abstract

**Background:**

Community engagement within biomedical research is broadly defined as a collaborative relationship between a research team and a group of individuals targeted for research. A Community Advisory Board (CAB) is one mechanism of engaging the community. Within genomics research CABs may be particularly relevant due to the potential implications of research findings drawn from individual participants on the larger communities they represent. Within such research, CABs seek to meet instrumental goals such as protecting research participants and their community from research-related risks, as well as intrinsic goals such as promoting the respect of participants and their community. However, successful community engagement depends on the degree to which CABs legitimately represent and engage with communities targeted for research. Currently, there is little literature describing the use of CABs in genomics research taking place in developing countries, and even less in the field of genomics research relating to mental illness. The aim of this article is to describe and consider the contributions made by a researcher-driven, population-specific CAB in a genomics of schizophrenia research project taking place in South Africa, from the perspective of the research team.

**Discussion:**

Four broad discussion topics emerged during the CAB meetings namely: 1) informed consent procedures, 2) recruitment strategies, 3) patient illness beliefs and stigma experiences, and 4) specific ethical concerns relating to the project. The authors consider these discussions in terms of their contributions to instrumental and intrinsic goals of community engagement.

**Summary:**

The CAB gave valuable input on the consent processes and materials, recruitment strategies and suggested ways of minimizing the potential for stigma and discrimination. All of these contributions were of an instrumental nature, and helped improve the way in which the research took place. In addition, and perhaps more importantly, the CAB made a unique and important contribution relating to intrinsic functions such as promoting the respect and dignity of research participants and their community. This was particularly evident in ensuring sensitivity and respect of the community’s traditional beliefs about schizophrenia and its treatment, and in this way promoting a respectful relationship between the research team and the participants.

## Background

Community engagement in biomedical research is broadly defined as a collaborative relationship between a research team and the population or group of individuals targeted for research [1, 2]. Such engagement has been encouraged as potentially valuable in promoting the protection and respect of research participants and the communities they belong to in biomedical research, and particularly in international collaborations taking place in developing countries [1–3]. Community engagement can take a number of forms, a Community Advisory Board (CAB) being a commonly used method. The aim of this article is to describe and consider the contributions made by a researcher-driver, population-specific CAB in a genomics of schizophrenia research project taking place in South Africa, from the perspective of the research team.

A CAB is typically defined as a group of people, representing the community targeted for research, who liaise between the research team and that community [4]. CABs have the potential of strengthening the science of the research study through improving informed consent materials and procedures, enhancing recruitment procedures and managing the research-related risks to participants and their community during the research process [5, 6], all of which can be understood as instrumental goals of community engagement. These instrumental goals can also have intrinsic value, for example CABs may act as advocates for the human rights of the community [4, 7], representing and advocating for community concerns about elements of the research process to the research team [4]. CABs also hold the potential of achieving intrinsic goals that focus on the promotion of trust and respect for the research participants and their community [5, 6], establishing relationships between the research team and the community [7], and providing the research team with insights into the unique social and cultural context within which a community operates [8]. Intrinsic goals may also include respect for a community’s traditional beliefs, understandings of illness, and help-seeking behaviours relating to treatment.

However, the success of this method depends on the degree to which a CAB legitimately represents and engages with the community targeted for research [5]. Key challenges in establishing CABs include defining the community being targeted for research, and achieving authentic and legitimate representation of this community through the CAB [5, 9]. A particular challenge relates to whether CABs are community- or researcher- initiated, and representative of very broad community groups, or rather more specific groups such as patient communities. Further challenges relate to how the CAB is formed. CABs may draw from a broad community model that provides representation of a cross section of a much larger community [8]. This model tends to be community initiated, promoting the autonomy of its members as advocate, and the rights of the community [8]. However broad community CABs may struggle with the challenge of generating resources and funding to support sustainability [8].

An alternative is a population-specific model that aims to represent the needs of a specific group of people [8]. This model tends to be initiated and funded by a particular research team to provide a voice for the targeted population [8, 9]. Population-specific CABs often contend with the challenge of maintaining the independence and autonomy of their members [8] on the one hand, and managing the input from community members who may be unfamiliar with biomedical research on the other [9]. Such input may offer novel perspectives that have not been considered by the research team, but if misinformed has the potential to compromise the quality of the science of the research [9]. A further challenge is the sustainability of such CABs, as funding is often project-related. Trust and rapport take time to build but project funding normally only lasts for a couple of years, after which the CAB may no longer be supported.

Within genomics research, community engagement through CABs may be particularly relevant considering the potential implications of research findings drawn from individual participants on their families and the larger communities and social groups they belong to [6]. Community engagement may protect against potential harms like discrimination and stigmatization arising out of research. Such harms could arise in genomics research when a particular community or social group is associated with a genetic predisposition towards a stigmatized illness [10]. Dignitary harms could arise as a result of a violation of rights or disrespectful treatment of participants and their community that is at odds with the cultural and traditional values of that community [11].

Genomics research into chronic, severe mental illness has the potential of placing participants at risk of: a) discrimination if confidentiality is breached or the group becomes known; b) negative psychological reactions as a result of becoming aware of a genetic risk; and c) confusion and distress about managing the sharing of this information with biological relatives [9, 12]. In addition, the families and communities these individuals belong to may be directly or indirectly impacted on through association and stigmatization as a result of this research [9, 12, 13]. Community engagement through CABs may be one means of addressing these concerns. In Africa, community engagement and the use of CABs within genomics research has received some attention but not in the field of psychiatry.

### ‘The genomics of schizophrenia in South African Xhosa People’ project

‘The Genomics of Schizophrenia in South African Xhosa People’ (SAX) project seeks to identify genes or mutations underlying predisposition to schizophrenia in the Xhosa population. Participants are presented with informed consent material in the Xhosa language that explains the SAX study. Recruiters talk through this material in Xhosa with participants and then evaluate participants’ level of understanding of the study using the University of California, San Diego Brief Assessment of Capacity to Consent questionnaire (UBACC) [14]. Participants complete a clinical assessment comprising the Structured Diagnostic Interview for DSM-IV Axis I Disorders (SCID-I), [15] a neurocognitive battery and additional psychosocial measures. Participants also provide blood samples for DNA testing. The study aims to recruit 1100 Xhosa people with a clinical diagnosis of schizophrenia or schizoaffective disorder and 1100 matched controls with no history of brain injury, substance-induced injury or history of psychosis, over a 5-year period. Currently in its third year, the study has recruited approximately 300 cases and 300 controls. The study is a member of the Human Heredity and Health in Africa Consortium (H3Africa) (http://www.h3africa.org) and receives funding from the National Institute of Mental Health (NIMH).

The Xhosa community fall historically within the group of racially segregated and oppressed South Africans discriminated against during Apartheid governance [16]. As a result the community bears the legacy of restricted access to healthcare, education and employment opportunities, that 20 years after the introduction of democracy in South Africa continues to perpetuate gross inequalities between population groups [16]. Severely mentally ill Xhosa patients diagnosed with schizophrenia are a small minority within this larger community.

## Methods

### Determining the most appropriate method of community engagement

In studies of severe mental disorders such as the SAX study, the targeted research participants belong to a vulnerable group, calling for special caution. The participants are likely to suffer stigma and discrimination, and may be struggling with poverty, unemployment and limited access to resources like healthcare and education. We introduced community engagement in the early stages of our project to assist with i) instrumental functions such as protecting participants and the Xhosa community from any research-related risks posed by the project, and ii) intrinsic functions such as promoting respect for people with schizophrenia, as well as for traditional Xhosa beliefs about schizophrenia and its treatment, during the recruitment process.

During community engagement defining the community and ensuring appropriate representation in the engagement process has proven to be a considerable challenge for researchers [9, 10]. The community is typically defined geographically to include individuals within a shared living area that have common lived experiences, shared situations and goals [6, 17]. However, our definition of community was primarily disease-based (i.e., targeting people with relevant experiences relating to schizophrenia, either as patients, caregivers, advocates or healthcare professionals) and secondarily culture based (i.e., targeting Xhosa people), requiring engagement with multiple stakeholders in representing this community. As opposed to other forms of community engagement, we considered a CAB the most appropriate means of engaging these representatives in on-going discussion together. In addition, a CAB offered a cost- and time-effective method of community engagement, that was well documented in the literature with helpful guidelines for implementation. A researcher-driven, population-specific CAB allowed the research team to develop a working relationship and rapport with a diverse range of representatives in managing research related concerns relating to the SAX study. No additional community engagement activities have been planned at this stage.

In genomics studies issues also arise in finding accessible language and means of explaining the complex concepts associated with genomics research (like genetics, genomics and data sharing) to potential participants and community representatives, particularly in developing countries where some individuals may have limited education and literacy [18]. Within the SAX study language is an important factor because all the participants are mother-tongue Xhosa speakers, and all engagement between participants and the research team is done in Xhosa. Therefore all informed consent materials have been translated into Xhosa. An important component of community engagement was to ensure that these Xhosa translations were meaningful and appropriate within the target sample.

### Selection of SAX CAB members

We sought individuals with lived experiences either as patients diagnosed with schizophrenia or as family members, caregivers or community volunteers working with patients diagnosed with schizophrenia. An advert was placed in local community newspapers requesting Xhosa community members to join the CAB. Local clinicians working with Xhosa people diagnosed with schizophrenia in state hospital contexts and known to the research team were invited by the team to participate in the CAB, as were hospital facility board members. Two Cape Town-based mental health advocacy groups were also approached by the team and invited to send representatives to join the CAB. Members joined the CAB as volunteers and did not receive any remuneration for their participation.

### Composition of the SAX CAB

The SAX CAB included ten community representatives: two male patients with schizophrenia who were active members of schizophrenia advocacy consumer groups; two female Xhosa residents of local communities within the recruitment catchment area, two female representatives from a local psychiatric hospital facility board who were familiar with many of the relevant clinical issues, two female members with leadership roles within mental health advocacy groups and one male and one female Xhosa clinician with extensive experience in psychiatry. All CAB members lived and/or worked within the geographical catchment area targeted for the initial recruitment stage of the project, had secondary or tertiary education and fell between 30 to 60 years of age.

The research team chose to include CAB members with understandings of research and clinical issues. We experienced this to be a particular strength of the CAB in that members were able to comment on research-related issues drawing from both their research experience and clinical insights having worked with Xhosa patients with schizophrenia, and being well acquainted with the community. However this may also have been a limitation in that this knowledge base steered the CAB in a particular direction.

A unique feature of the way in which this CAB was structured is that it also included members of the research team who actively participated in CAB discussions. In total seven members of the research team participated in the CAB meetings: two male primary investigators (RR and DS), two male project managers (AB and GS) and three male Xhosa psychiatric nurses performing fieldwork (MM, SM and ON). The psychiatric nurses played a dual role as research team members and as Xhosa people who worked with Xhosa patients diagnosed with schizophrenia. They brought ethical concerns and challenges experienced in the field to the CAB meetings for discussion.

Having the research team present during the CAB meetings arguably had an influence over the group meetings. Firstly it’s likely that the research team members held an elevated status in the group as researchers and mental health professionals that may have prompted other members of the CAB to behave in socially desirable ways towards these members. Their presence and participation in the group arguably influenced the topics and content of discussion that took place during CAB meetings. Second, it’s likely that SAX team members were placed in conflicting roles on occasions when having to advocate for i) the interests of the SAX study, ii) the interests of Xhosa people with schizophrenia and iii) the interests of the Xhosa community. However despite these limitations the primary goal of this research team driven CAB was to develop a working relationship and rapport between the SAX research team and a diverse range of stakeholders representing patients with schizophrenia as well as the Xhosa community. Having a combination of community representatives and SAX research team members engaged in CAB meetings seemed a pragmatic way of developing this relationship.

### Structure and process of CAB meetings

The CAB was constituted in July 2013 and meetings took place every three to four months resulting in a total of four CAB meetings. The research team set the agenda for each meeting, which typically included an update on the status of the study and current ethical challenges that had emerged since the last CAB meeting. Each agenda item was described by a representative of the research team, providing opportunity for questions and clarifications by CAB members, and then opened for broader discussion. During meetings, more introverted CAB member were particularly encouraged to comment in order to guard against stronger personalities dominating the group discussion. Meetings typically lasted 60–90 min. On average, of the seventeen CAB members (ten community representatives and seven research team members) fourteen members attended each CAB meeting. Absent members included both research team members and community representatives.

### Data collection and analysis

The intention of the authors was to develop an initial exploratory manuscript that described the researchers’ experiences of working with a researcher-driven, population-specific CAB. As a result data collection concentrated on the minutes generated during the CAB meetings, supplemented by observations and personal notes taken by research team members during these meetings. Minutes of the four CAB meetings occurring between July 2013 and August 2014, were taken by the first author (MC) who was neither a member of the research team nor the CAB. Permission to record these CAB discussions for possible publication was obtained in the first CAB meeting. These minutes comprised 12 pages of summarized points of discussion raised during the meetings. This text, together with personal observations and notes taken during the CAB meetings by other research team members were carefully read by MC in order to understand the topics of discussion as well as the concerns and contributions of the CAB. Four broad Meeting minutes were circulated to the CAB members for comment and review, in order to ensure fair representation of the discussions.

The research team members participating in the CAB also contributed to the writing up of this article drawing from their tacit knowledge of the Xhosa community, the community engagement experience, and how CAB contributions were being integrated into the SAX study at the recruitment phase. One limitation of this method was that it excluded additional contextual information from CAB members or Xhosa people with schizophrenia who the CAB was developed to represent.

## Findings and discussion

Four broad discussion areas emerged during the CAB meetings. These included 1) informed consent procedures, 2) recruitment strategies, 3) patient illness beliefs and stigma experiences and 4) specific ethical concerns relating to the project.

### Informed consent procedures

One of the key challenges in genomics research in Africa is obtaining valid informed consent from research participants [18]. Research participants should be adequately informed about the study, have an appropriate level of understanding about the science and research methods of the study, and give voluntary consent to participate [18–20]. However, low literacy and education levels, combined with language and cultural differences make this a challenging process and community engagement has been suggested as a helpful mechanism in achieving this instrumental goal [18, 21]. In our project, these challenges were further compounded in that the written Xhosa language is not standardized, making conceptually and linguistically equivalent language translations from English to Xhosa difficult [21].

In reviewing the information sheets and informed consent documents that had been prepared for the study, the CAB made three suggestions. Firstly, CAB members acknowledged the complexity of the scientific and medical terminology used in genomics research, and emphasized the need for fieldworkers involved in the recruitment process to use simple, accessible language and visual aides in explaining the project. Secondly, CAB members noted the difficulty in finding appropriate terminology to refer to schizophrenia in the Xhosa language. Initially one of the project managers had suggested “iingxaki ezenziwa lufuthe lwemeko kunye neengxaki zeengcinga” (emotional or thought problems). However some CAB members considered this too broad a description. Additional suggestions included “ukuphazamiseka kwengqondo” (general disturbances of the mind) or “ukushiywa zingqondo” (losing your mind). These three examples illustrate to some extent the difficulty in finding agreement about this term in the Xhosa language. It was decided to use the word “schizophrenia” as this was the most clear and least offensive, and rely on fieldworkers to explain the term to the participants. Thirdly, CAB members suggested pragmatic changes to the informed consent material that included a separate document for consenting to HIV testing, and the correction of spelling and grammar mistakes in the Xhosa translations of the informed consent materials.

In these discussions the CAB recognized the challenges relating to the complexity of genomics terminology. While these have been documented in the literature [19, 20, 22] highlighting these challenges reiterated the need for intensive training of fieldworkers about the science behind the project, in order to adequately equip them with the skills necessary to explain the project in language understandable to potential participants. Feedback from the CAB also allowed the research team to gauge the accessibility and appropriateness of their informed consent material before introducing it to potential research participants. Through these interactions with the CAB the research team was able to gain feedback that reiterated their own understandings about challenges that could arise in the community during recruitment, and to develop and discuss possible ways of managing these challenges. However few solutions were found to these challenges.

### Recruitment strategies

An additional instrumental goal of community engagement within genomics research in African settings is improved recruitment strategies, particularly in contexts where permission is required by community gatekeepers like chiefs and elders [22, 23]. Such engagement establishes trust and rapport between the research team and the community. Our study recruited patients in urban areas and we had not initially identified who the community gatekeepers in our setting would be. However, during discussions about recruitment, the CAB suggested that the research team co-ordinate their recruitment through community hospital nurses who were familiar with the communities in the catchment areas targeted for research. This strategy was suggested as a way of utilizing the trust and rapport already established by these nurses with the community, in order to assist in recruitment. CAB members also noted that some research participants may move to different residential addresses or new communities and geographical areas. They therefore encouraged research assistants to obtain multiple contacts for research participants. These were helpful, practical suggestions that assisted the research team in planning their recruitment strategies. These CAB contributions highlighted how individuals less familiar with biomedical research processes were able to make valuable contributions to recruitment procedures.

### Patient illness beliefs and stigma experiences

Biomedical research takes place within a wider social, economic, political and cultural context that frames research participants’ understandings of medicine, medication, their illness experiences and the research process [12, 21, 23–25]. Community engagement aims at building awareness and sensitivity to these unique contextual factors at play within the research process, promoting respect for and understanding of the community, and in this way promoting a respectful relationship between the research team and the participants [12]. The CAB highlighted that schizophrenia could be perceived within Xhosa communities as a bewitchment that requires traditional healing. Therefore some Xhosa patients may be unfamiliar with seeking help for their symptoms from a hospital or clinic where they are presented with illness explanations that challenge their currently held traditional beliefs about their illness. In other words, potential research participants could struggle with a biomedical explanation of schizophrenia. CAB members voiced concerns regarding poor mental health literacy about schizophrenia within Xhosa communities that included 1) unfamiliarity with the term schizophrenia and biomedical explanations of the illness, and 2) feelings of shame experienced amongst family members for being related to a patient diagnosed with schizophrenia. CAB members commented that poor formal education, low socio-economic status, and rural living conditions restricting access to psycho-educational material about severe mental illness all contributed to the stigma experienced by patients with schizophrenia.

In response to these concerns the CAB recommended that a local community newsletter, produced and distributed by a mental health advocacy group promoting community mental healthcare issues, be used as a forum for updating the community about the project. The intention would be to use the newsletter to disseminate information about a biomedical explanation of schizophrenia to the community. The newsletter was also suggested as a platform to disseminate findings from the project as they became available. In addition, CAB members questioned how the mental health needs of the control group were being managed within the study and recommended referral channels and services that would assist these individuals in managing both emotional and social problems.

The insights and suggestions shared by the CAB relating to explanatory models of mental illness and schizophrenia within Xhosa communities, as well as patient stigma experiences have been documented in South African mental health literature [26–30]. However recognition of these explanatory models and stigma experiences by the CAB emphasized the need for culture-sensitive training of research assistants involved in recruitment within the target communities. This training required a focus on understanding and being sensitive to the beliefs held by the Xhosa community about severe mental illness and schizophrenia, as well as the stigma experiences of patients with schizophrenia. This understanding would assist the fieldworkers in establishing a working rapport with potential research participants, and contribute towards upholding the respect and dignity of the Xhosa community and their traditional beliefs.

The CAB’s knowledge contributions regarding indigenous Xhosa explanatory illness models and patient stigma experiences are well established in the South African mental health literature. However CAB opinions and suggestions about how to manage this knowledge strengthened the research team’s understandings of these challenges. In this way the CAB promoted the respect and dignity of the community’s cultural and traditional beliefs and in so doing promoted a respectful relationship between the research team and the participants.

### Specific concerns relating to the research project

In accordance with the literature, while the use of a CAB is a popular mechanism of community engagement, the employment of community members as fieldworkers is an additional way of taking the contextual factors and social relationships at play within a community into account during research [31]. These community members have cultural insights into traditional community beliefs and cultural norms, speak the language and are considered insiders and as such can assist with trust and rapport building [17]. The CAB included Xhosa psychiatric nurses employed as fieldworkers. These nurses played a dual role as research team members as well as Xhosa people, representative of the community from which Xhosa patients with schizophrenia were being recruited. Having these nurses actively participate in the CAB allowed for a unique exchange of opinions and suggestions.

The research team brought two specific ethical concerns to the CAB for comment. The first involved the sharing and secondary analysis of genomic material and related data. This complex issue had been raised as an ethical concern by the Research Ethics Committee (REC) at our institution. Specific challenges that were identified related to difficulties in translating these concepts and understandings into Xhosa, challenges in ensuring that participants understood their right to choose whether to allow the sharing of their genomic material and data, and understanding of the implications of choosing to donate their samples for future research. Presentation of these issues to the CAB resulted in interesting discussion and debate. CAB members felt that people with schizophrenia, despite low health literacy and particular explanatory models, were able to give valid informed consent to issues relating to data sharing and secondary analysis. The CAB was supportive of the view that collaborative science between developed and developing countries was needed, and similarly that international genomics research should also involve people of Xhosa ancestry. In addition CAB members felt that if other, non-schizophrenia patients could give consent for sample sharing for unspecified future research, there was no reason to restrict psychiatry patients in their choices. Some members felt that restricting this choice could be experienced as stigmatizing to patients with schizophrenia, whilst others suggested it could cause individuals unnecessary distress in not knowing how their genomic material and data was going to be used. The CAB consensus was that individual research participants should be given the choice to decide whether or not to consent to secondary sample and data sharing.

The research team also asked the CAB to comment on a challenge arising in the recruitment of control participants. In accordance with REC recommendations each individual who participated in the project received R150 for their time and effort. However, considering the tremendous socio-economic inequalities that currently exist in South Africa, with the average annual income for a black South African estimated at R21 075 in 2010 (approximately R1760/month) [16], compensation of R150 for one day of participation in a research study is a substantial incentive. As a result word quickly spread about the Genomics of Schizophrenia recruitment process, and in the first few weeks of the study fieldworkers were overwhelmed by willing control participants who had been encouraged by friends and family to enroll in the study in order to receive financial compensation for their blood samples. The research team was uncertain as to how to manage this and sought advice from the CAB.

One suggestion made by the CAB was to change the sites where the controls were being recruited to community centres and churches. However other CAB members noted that these settings may be more difficult to contain. An additional suggestion was to request that the community hospital-based nurses at each site be the primary channel of referral. These nurses would be familiar with the patients and able to guide the referral of these patients to the research assistants.

In these examples the CAB provided a platform for the research team to bring ethical concerns emerging in the field to the CAB for comment and debate. The CAB in turn provided suggestions about how these challenges could be managed. Some of these suggestions were helpful and informative, while others presented their own ethical challenges. For example the CAB’s suggestion of addressing the high rate of control volunteers by screening through local community nurses may result in favoritism and selection bias. While CAB members did not provide resolution to these ethical concerns, they provided a space where the research team could bring these particular ethical concerns for discussion. They also provided insights into different opinions that may surface within the community relating to these issues, equipping the research team with additional community understandings that could assist in managing these potential ethical concerns.

## Summary

The initial community engagement goals of this SAX CAB were to i) protect participants and the Xhosa community from any research-related risks posed by the project through instrumental goals, and to ii) promote respect for Xhosa people with schizophrenia and their traditional Xhosa beliefs through intrinsic goals. When reflecting on the discussions arising during the CAB meetings two main points emerge.

First, the CAB made helpful contributions towards instrumental goals such as improved informed consent materials, recruitment strategies and protecting research participants and the community from research-related risks. On one hand it could be argued that many of these contributions may also be obtained through existing mental health literature, consultation with other researchers familiar with the research settings and the target population, and Xhosa clinicians working with these patients. However having a single group of individuals, representative of the target community, to consult on instrumental goals of the research process was an efficient and helpful way of validating the researchers’ decisions around these processes. The pragmatic suggestions made by the CAB indicate that individuals unfamiliar with biomedical research can make valuable contributions to key research processes. CAB members may however be influenced by social desirability, particularly when research team members are actively engaged in the CAB. A future empirical study evaluating the contributions made by the SAX CAB, combining the perspectives of both the community and research team representatives would be valuable.

Second, our community engagement activities seemed to make a valuable contribution towards the intrinsic goal of respecting the research participants and their community within the research process. This was particularly evident in ensuring sensitivity to and respect of the community’s traditional beliefs about schizophrenia and its treatment. Speaking from the perspective of the research team, we feel that the CAB promoted a respectful relationship between the research team and the participants. Constituted by individuals with key knowledge of the Xhosa community, the CAB meetings provided a space for the research team to engage with these representatives about culturally sensitive engagement with Xhosa patients recruited for the study. The result, we feel, was that community engagement established a more respectful means of approaching the community and prospective participants through an openness to understand their explanations of schizophrenia and treatment, instead of merely imposing a biomedical view onto the participants.

## References

[1] Council for International Organizations of Medical Sciences (2002). International Ethical Guidelines for Biomedical Research Involving Human Subjects. Bull Med Ethics.

[2] Nuffield Council on Bioethics (2002). The ethics of research related to healthcare in developing countries.

[3] Tindana PO, Singh JA, Tracy CS, Upshur RE, Daar AS, Singer PA (2007). Grand challenges in global health: community engagement in research in developing countries. PLoS Med.

[4] Strauss RP, Sengupta S, Quinn SC, Goeppinger J, Spaulding C, Kegeles SM (2001). The Role of Community Advisory Boards: Involving Communities in the Informed Consent Process. Am J Public Health.

[5] Angwenyi V, Kamuya D, Mwachiro D, Kalama B, Marsh V, Njuguna P (2014). Complex realities: community engagement for a paediatric randomized controlled malaria vaccine trial in Kilifi, Kenya. Trials.

[6] Participants in the Community Engagement and Consent Workshop, Kilifi, Kenya, March 2011 (2013). Consent and community engagement in diverse research contexts: reviewing and developing research and practice. J Empir Res Hum Res Ethics.

[7] Quinn SC (2004). Ethics in Public Health Research: Protecting Human Subjects: the Role of Community Advisory Boards. Am J Public Health.

[8] Morin SF, Morfit S, Maiorana A, Aramrattana A, Goicochea P, Mutsambi JM (2008). Building community partnerships: case studies of Community Advisory Boards at research sites in Peru, Zimbabwe, and Thailand. Clin Trials.

[9] DuBois J, Bailey-Burch B, Bustillos D, Campbell J, Cottler L, Fisher C (2011). Ethical issues in mental health. Curr Opin Psychiatry.

[10] Sharp RR, Foster MW (2000). Involving Study Populations in the Review of Genetic Research. J Law Med Ethics.

[11] de Vries J, Jallow M, Williams TN, Kwiatkowski D, Parker M, Fitzpatrick R (2012). Investigating the potential for ethnic group harm in collaborative genomics research in Africa: Is ethnic stigmatisation likely?. Soc Sci Med.

[12] Sharp RR, Foster MW (2002). Community involvement in the ethical review of genetic research: lessons from American Indian and Alaska Native populations. Environ Health Perspect.

[13] Hoop JG (2008). Ethical considerations in psychiatric genetics. Harv Rev Psychiatry.

[14] Jeste DV, Palmer BW, Appelbaum PS, Golshan S, Glorioso D, Dunn LB (2007). A new brief instrument for assessing decisional capacity for clinical research. Arch Gen Psychiatry.

[15] First MB, Spitzer RL, Gibbon M, Williams JB (2012). Structured Clinical Interview for DSM-IV® Axis I Disorders (SCID-I), Clinician Version, Administration Booklet.

[16] South African Institute of Race Relations. South African Survey Online. 2003. [www.irr.org.za]. Accessed 15 Jan 2014.

[17] Kamuya D, Marsh V, Kombe F, Geissler W, Molyneux S (2013). Engaging communities to strengthen research ethics in low-income settings: selection and perceptions of members of a network in coastal Kenya. Dev World Bioeth.

[18] de Vries J, Bull S, Doumbo O, Ibrahim M, Mercereau-Puijalon O, Kwiatkowski D (2011). Ethical issues in human genomics research in developing countries. BMC Med Ethics.

[19] Tindana P, Bull S, Amenga-Etego L, de Vries J, Aborigo R, Koram K (2012). Seeking consent to genetic and genomic research in a rural Ghanaian setting: A qualitative study of the MalariaGEN experience. BMC Med Ethics.

[20] Marshall P, Adebamowo C, Adeyemo A, Ogundiran T, Strenski T, Zhou J (2014). Voluntary participation and comprehension of informed consent in a genetic epidemiological study of breast cancer in Nigeria. BMC Med Ethics.

[21] Swartz L (1998). Culture and mental health: a southern African view.

[22] Marsh VM, Kamuya DM, Mlamba AM, Williams TN, Molyneux SS (2010). Experiences with community engagement and informed consent in a genetic cohort study of severe childhood diseases in Kenya. BMC Med Ethics.

[23] Benatar SR (2002). Reflections and recommendations on research ethics in developing countries. Soc Sci Med.

[24] Benatar SR, Singer PA (2000). A new look at international research ethics. BMJ.

[25] Benatar SR, Singer PA (2010). Responsibilities in international research: a new look revisited. J Med Ethics.

[26] Mbanga NI, Niehaus DJH, Mzamo NC, Wessels CJ, Allen A, Emsley RA (2002). Attitudes towards and beliefs about schizophrenia in Xhosa families with affected probands. Curationis.

[27] Niehaus DJH, Oosthuizen P, Lochner C, Emsley RA, Jordaan E, Mbanga NI (2004). A culture-bound syndrome ‘amafufunyana’and a culture-specific event ‘ukuthwasa’: differentiated by a family history of schizophrenia and other psychiatric disorders. Psychopathology.

[28] Sorsdahl KR, Flisher AJ, Wilson Z, Stein DJ (2010). Explanatory models of mental disorders and treatment practices among traditional healers in Mpumulanga, South Africa. Afr J Psychiatry.

[29] Hugo CJ, Boshoff D, Traut A, Zungu-Dirwayi N, Stein D (2003). Community attitudes toward and knowledge of mental illness in South Africa. Soc Psychiatry Psychiatr Epidemiol.

[30] Sorsdahl KR, Stein DJ (2010). Knowledge of and stigma associated with mental disorders in a South African community sample. J Nerv Ment Dis.

[31] Kamuya DM, Theobald SJ, Munywoki PK, Koech D, Geissler WP, Molyneux SC (2013). Evolving friendships and shifting ethical dilemmas: fieldworkers’ experiences in a short term community based study in Kenya. Dev World Bioeth.

